# Supplementary material for: mTORC1 regulates cell survival under glucose starvation through 4EBP1/2-mediated translational reprogramming of fatty acid metabolism
Source: Nat Commun. 2024 May 14;15:4083. doi: 10.1038/s41467-024-48386-y (PMC11094136; doi:10.1038/s41467-024-48386-y)
Supplement: Supplementary file 1 — Supplementary Information [file 41467_2024_48386_MOESM1_ESM.pdf]

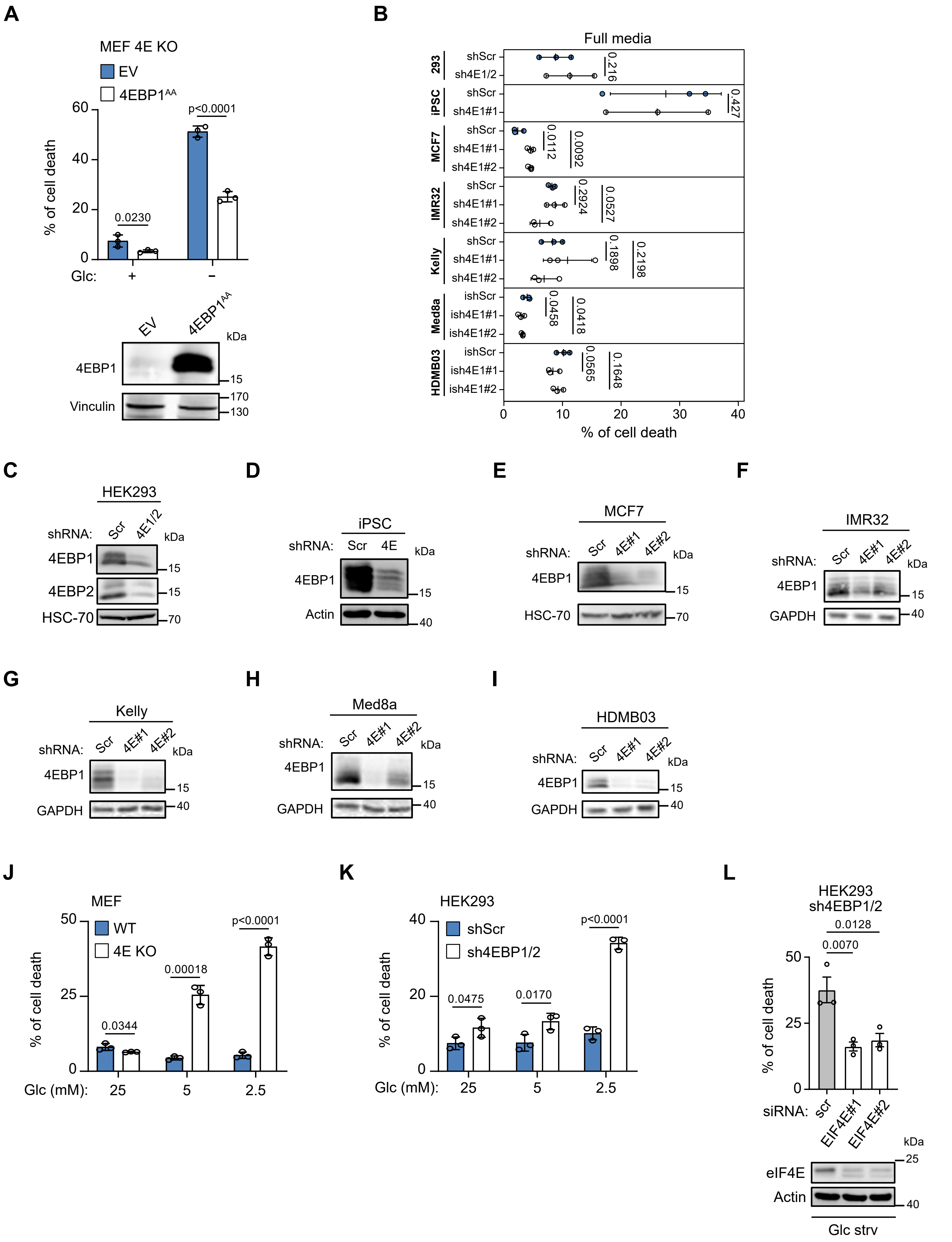

A
MEF 4E KO
EV
4EBP1AA
% of cell death
0.0230
p<0.0001
Glc: + −
EV
4EBP1AA
kDa
4EBP1
Vinculin
15
170
130
B
Full media
293
iPSC
MCF7
IMR32
Kelly
Med8a
HDMB03
shScr
sh4E1/2
sh4E1#1
sh4E1#2
ishScr
ish4E1#1
ish4E1#2
0.216
0.427
0.0112
0.0092
0.2924
0.0527
0.1898
0.2198
0.0458
0.0418
0.0565
0.1648
% of cell death
C
HEK293
shRNA: Scr 4E1/2
4EBP1
4EBP2
HSC-70
D
iPSC
shRNA: Scr 4E
Actin
E
MCF7
shRNA: Scr 4E#1 4E#2
F
IMR32
GAPDH
G
Kelly
H
Med8a
I
HDMB03
J
MEF
WT
4E KO
0.0344
0.00018
p<0.0001
Glc (mM): 25 5 2.5
K
HEK293
shScr
sh4EBP1/2
0.0475
0.0170
p<0.0001
L
HEK293 sh4EBP1/2
0.0128
0.0070
siRNA: scr EIF4E#1 EIF4E#2
eIF4E
Actin
Glc strv

Supplementary Figure 1. The mTORC1 substrates 4EBP1/2 support cell survival under glucose starvation

(A) Control (EV) and stable $4EBP1^{AA}$ overexpressing 4E KO MEFs were grown in complete media or starved for glucose (Glc) for 48 hrs. Cell death was measured by PI staining and flow cytometry. The level of the indicated proteins was analyzed by immunoblotting. (B) Control (shScr) and stable 4EBP1 and 4EBP2 knock down (sh4EBP1/2) HEK293 cells, or shScr and stable 4EBP1 knock down (sh4EBP1) iPSC, MCF7, IMR-32, and Kelly, or inducible control (ishScr) and stable 4EBP1 knock down (ish4EBP1) Med8a and HD-MB03 cells were grown in complete media. Med8a and HD-MB03 cells were treated with 1 μg/ml doxycycline for 72 hrs. Cell death was measured as in (A). (C-I) Cells from (B) were analyzed by immunoblotting for the levels of the indicated proteins. Representative immunoblots of two independent experiments are shown. (J) WT and 4E KO MEFs were grown in complete medium or medium containing the indicated concentrations of glucose for 48 hrs. Cell death was measured as in (A). (K) ShScr and sh4EBP1/2 HEK293 cells were grown in complete medium or medium containing the indicated concentrations of glucose for 48 hrs. Cell death was measured as in (A). (L) ShScr and sh4EBP1/2 HEK293 cells were transfected with control siRNA (scr) or siRNAs targeting *EIF4E* and grown in glucose starved medium (Glc strv) for 48 hrs. Cell death was analyzed as in (A) and the level of the indicated proteins was analyzed by immunoblotting. Data are shown as the mean ± SD. Statistics: unpaired one-sided (A, J, K, L) or two-sided (B) Student's *t* test (b); n=3 independent experiments for A, B, J, K, L. Source data are provided as a Source Data file.

Supplementary Figure 2

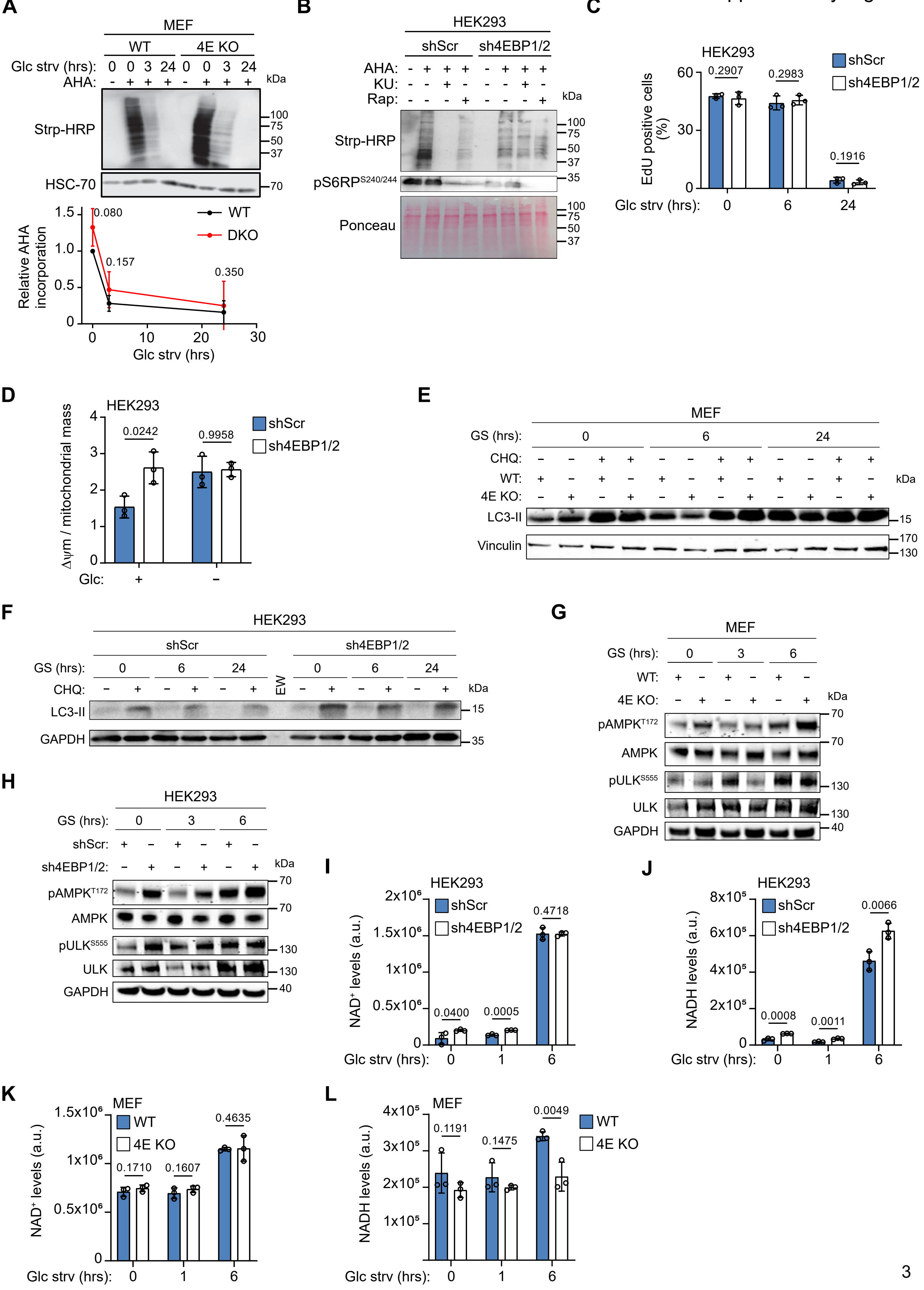

Supplementary Figure 2. 4EBP1/2 do not control overall protein synthesis, proliferation, mitochondrial activity or autophagy under glucose starvation

(A) The indicated cells were grown in complete medium or glucose starved (Glc strv) for the indicated times and labeled with azidohomoalanine (AHA). Levels of AHA-labelled proteins were detected by immunoblotting. (B) The indicated cells were grown in complete media, treated or not with rapamycin (Rapa) or KU-0063794 (KU), and labeled with azidohomoalanine (AHA). Levels of AHA-labelled proteins were detected by immunoblotting with a streptavidin conjugate. Representative immunoblots of three independent experiments are shown for A, B. (C) The indicated cells were grown in complete medium or glucose starved (Glc strv) for the indicated times and labeled with EdU. The percentage of EdU-positive cells was analyzed by flow cytometry. (D) The indicated cells were grown in complete medium or glucose (Glc) starved for 24 hrs. Mitochondrial membrane potential ($\Delta\psi m$) was measured with TMRE staining and mitochondrial mass with MitoTracker™ Green FM and analyzed by flow cytometry. (E, F) The indicated cells were grown in complete medium or glucose starved (GS) for the indicated times with or without chloroquine (CHQ). Cell lysates were analyzed by immunoblotting using antibodies against the indicated proteins. Representative immunoblots of three independent experiments are shown. (G, H) The indicated cells were grown in complete medium or glucose starved (GS) for the indicated times. Cell lysates were analyzed by immunoblotting using antibodies against the indicated proteins. Representative immunoblots of three independent experiments are shown. (I-L) The indicated cells were grown in complete medium or glucose starved (glc strv) for the indicated times and NAD^+ or NADH levels were measured. Data are shown as the mean ± SD. Statistics: unpaired one-sided (C, D) or two-sided (I, J, K, L) Student's *t* test; n=3 independent experiments for A, B, C, D, I, J, K, L. Source data are provided as a Source Data file.

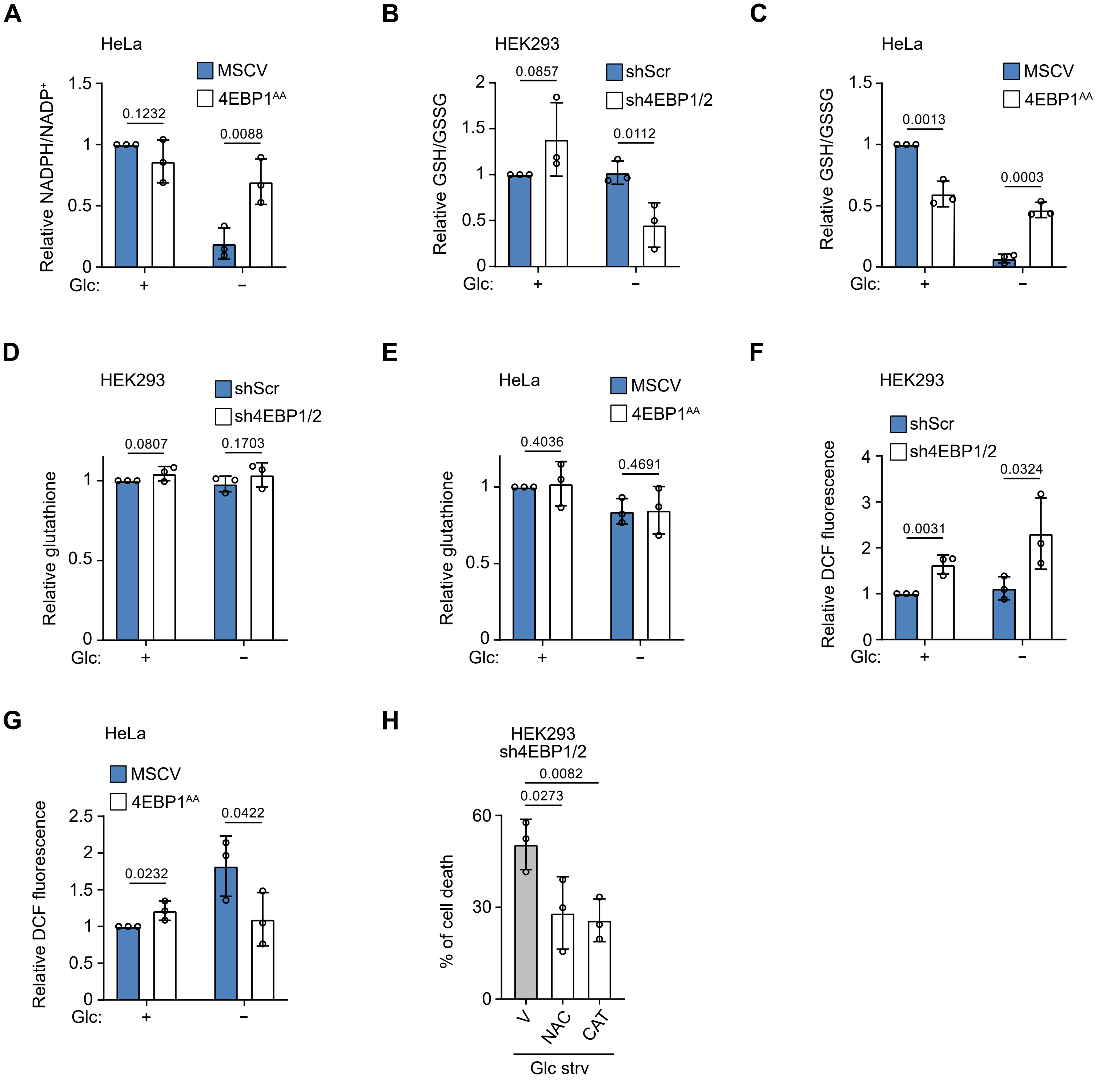

Supplementary Figure 3. 4EBP1/2 regulate intracellular redox balance under glucose starvation

(A) MSCV and stable 4EBP1^{AA} overexpressing HeLa cells were grown in complete medium or glucose (Glc) starved for 24 hrs, and NADP^{+} and NADPH levels were measured. (B, C) Control (shScr) and sh4EBP1/2 HEK293 (B), or MSCV and stable 4EBP1^{AA} overexpressing HeLa cells (C) were grown in complete medium or glucose (Glc) starved for 24 hrs, and reduced and total glutathione were measured and expressed as the ratio of reduced (GSH) to oxidized (GSSG) glutathione. (D, E) ShScr and sh4EBP1/2 HEK293 cells (D), or MSCV or 4EBP1^{AA} overexpressing HeLa cells (E) were grown in complete medium or glucose (Glc) starved for 24 hrs, and total glutathione was measured. (F, G) ShScr and sh4EBP1/2 HEK293 (F), or MSCV and stable 4EBP1^{AA} overexpressing HeLa cells (G) grown in complete medium or glucose (Glc) starved for 24 hrs were labelled with CM-DCFDA and analyzed by flow cytometry. (H) Sh4EBP1/2 HEK293 cells were grown in glucose starved medium (Glc strv) and treated with vehicle (V), N-acetyl cysteine (NAC) or Catalase (CAT) for 48 hrs. Cell death was measured by PI staining and flow cytometry. Data are shown as the mean ± SD. Statistics: unpaired one-sided Student's *t* test (A, B, C, D, E, F, G, H); n=3 independent experiments for A, B, C, D, E, F, G, H. Source data are provided as a Source Data file.

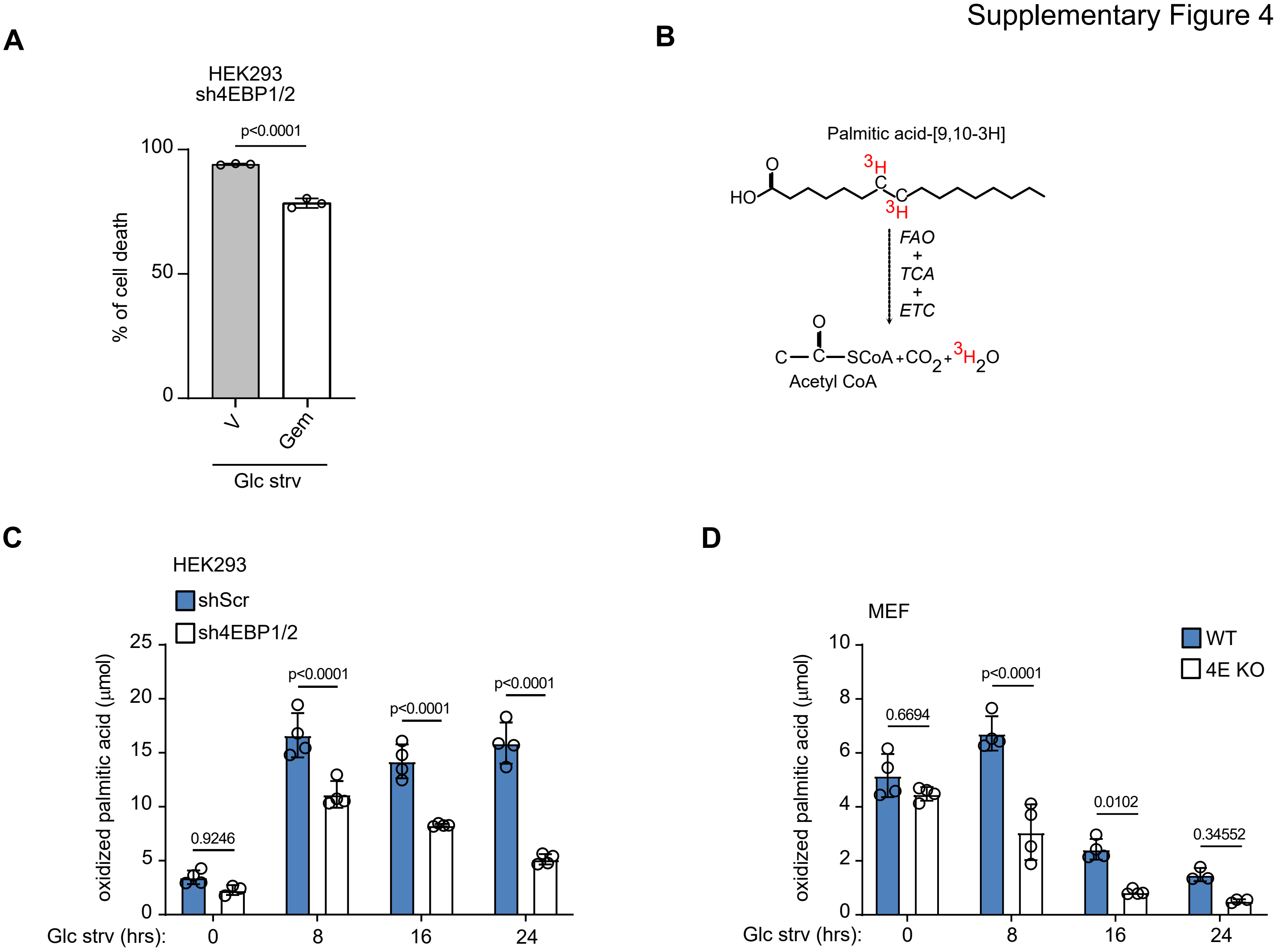

Supplementary Figure 4. 4EBP1/2 promote fatty acid oxidation under glucose deprivation

(A) Sh4EBP1/2 HEK293 cells were grown in glucose starved medium (Glc strv) with vehicle (V) or with gemcitabine (Gem) for 48 hrs. Cell death was measured by PI staining and flow cytometry. n=3 independent experiments. (B) Scheme of the [^{3}H] palmitate labeling assay to measure fatty acid oxidation activity. FAO: fatty acid oxidation, TCA: tricarboxylic acid cycle, ETC: electron transport chain. (C, D) Control (shScr) and sh4EBP1/2 HEK293 cells (C), or WT and 4EBP1/4EBP2 DKO (4E KO) MEF (D), grown in complete medium or glucose (Glc) starved for 24 hrs were labeled with [^{3}H] palmitate in the last 6 hrs. [^{3}H] was measured in conditioned media using a scintillation counter and normalized to total protein levels and by the percentage of [^{3}H] collected. n=3-4 independent experiments. Data are shown as the mean ± SD. Statistics: unpaired one-sided Student's *t* test (A), two way ANOVA (C, D). Source data are provided as a Source Data file.

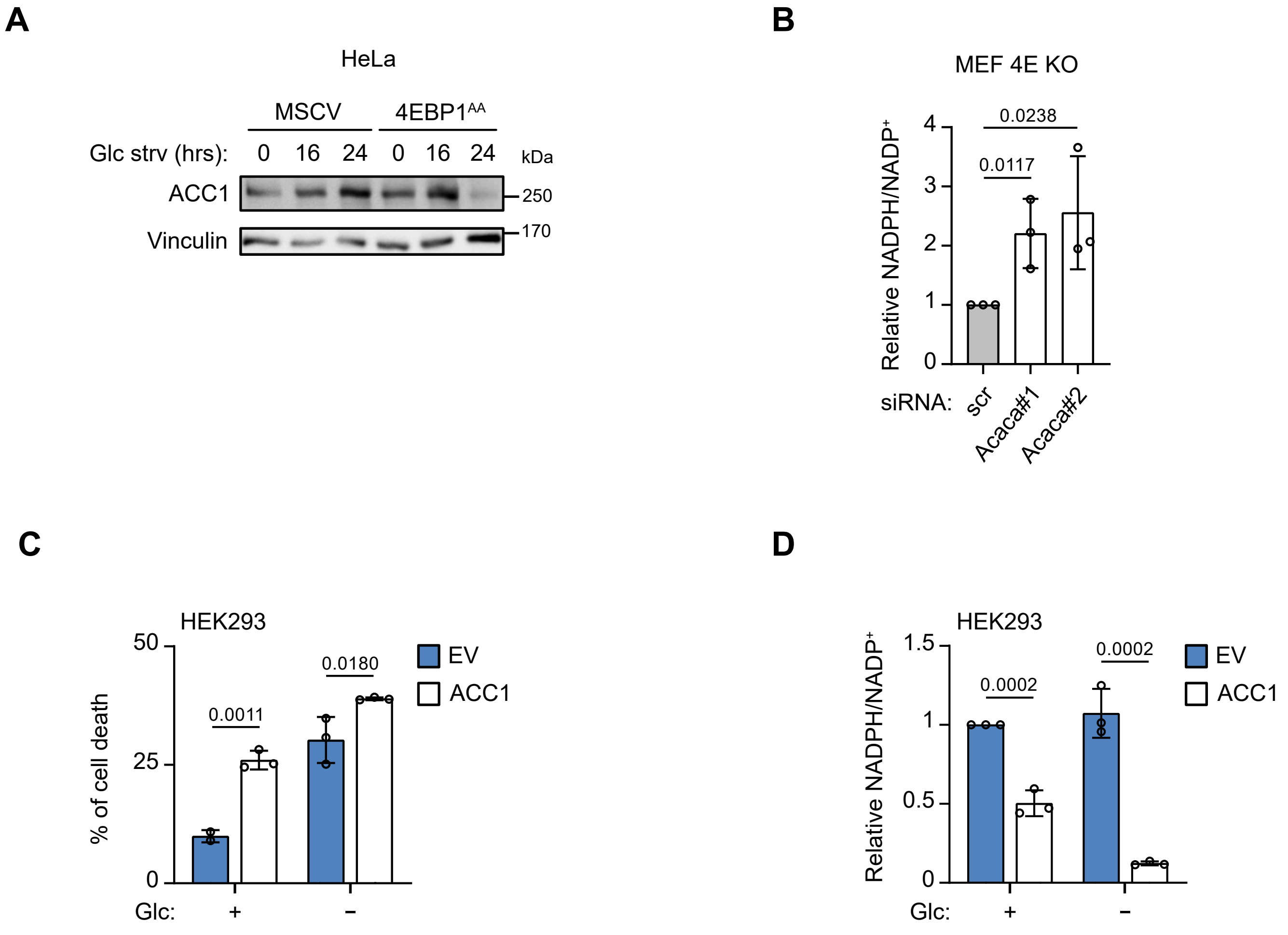

Supplementary Figure 5. The 4EBP1-ACC1 axis regulates cell survival and NADPH levels under glucose starvation (A) MSCV or 4EBP1^{AA} overexpressing HeLa cells were grown in complete medium or glucose starved (Glc strv) for the indicated times and analyzed by immunoblotting using antibodies against ACC1 and Vinculin as reference. Representative immunoblots of three independent experiments are shown. (B) 4E KO MEF were transfected with control siRNA (scr) or siRNAs targeting *Acaca*, grown in glucose starved medium (Glc strv) for 48 hrs and NADP$^+$ and NADPH levels were measured. (C) HEK293 cells transfected with empty vector (EV) or with an ACC1 overexpressing vector were grown in complete medium or glucose (Glc) starved for 48 hrs. Cell death was measured by PI staining and flow cytometry. (D) HEK293 cells transfected with empty vector (EV) or with an ACC1 overexpressing vector were grown in complete medium or glucose (Glc) starved for 24 hrs and NADP$^+$ and NADPH levels were measured. Data are shown as the mean ± SD. Statistics: unpaired one-sided Student's *t* test (B, C, D); n=3 independent experiments for B, C, D. Source data are provided as a Source Data file.

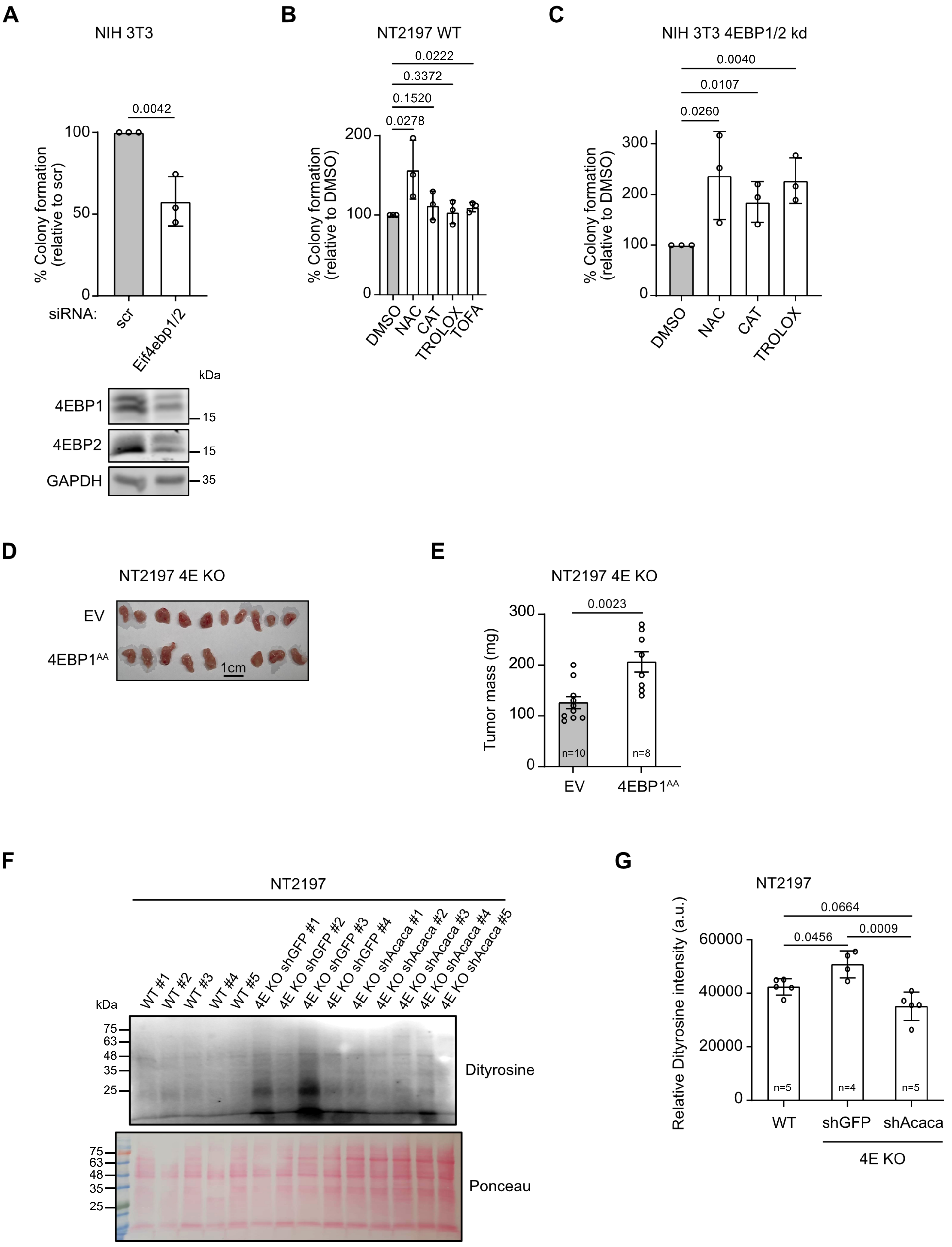
A
NIH 3T3
0.0042
% Colony formation (relative to scr)
100
50
0
siRNA:
scr
Eif4ebp1/2
kDa
4EBP1
15
4EBP2
15
GAPDH
35
B
NT2197 WT
0.0222
0.3372
0.1520
0.0278
200
100
0
% Colony formation (relative to DMSO)
DMSO
NAC
CAT
TROLOX
TOFA
C
NIH 3T3 4EBP1/2 kd
0.0040
0.0107
0.0260
300
200
100
0
% Colony formation (relative to DMSO)
DMSO
NAC
CAT
TROLOX
D
NT2197 4E KO
EV
4EBP1AA
1cm
E
NT2197 4E KO
0.0023
300
200
100
0
Tumor mass (mg)
n=10
n=8
EV
4EBP1AA
F
NT2197
WT #1
WT #2
WT #3
WT #4
WT #5
4E KO shGFP #1
4E KO shGFP #2
4E KO shGFP #3
4E KO shGFP #4
4E KO shAcaca #1
4E KO shAcaca #2
4E KO shAcaca #3
4E KO shAcaca #4
4E KO shAcaca #5
kDa
75
63
48
35
25
Dityrosine
75
63
48
35
25
Ponceau
G
NT2197
0.0664
0.0456
0.0009
60000
40000
20000
0
Relative Dityrosine intensity (a.u.)
n=5
n=4
n=5
WT
shGFP
shAcaca
4E KO

Supplementary Figure 6. 4EBP1/2 support oncogenic transformation by controlling the redox balance and ACC1
(A) NIH 3T3 transfected with control (scr), or *Eif4ebp1* and *Eif4ebp2* targeting siRNAs (*Eif4ebp1/2*) were grown in soft agar for 21 days. Colonies and single cells were counted, and colony formation efficiency was calculated. The level of the indicated proteins was analyzed by immunoblotting. (B) 4EBP1/2 WT NT2197 cells were grown in soft agar for 21 days and treated with DMSO, NAC, CAT, TROLOX or TOFA. Colonies and single cells were counted, and colony formation efficiency was calculated. (C) NIH 3T3 transfected with *Eif4ebp1* and *Eif4ebp2* targeting siRNAs (4EBP1/2 kd) were grown in soft agar for 21 days and treated with DMSO, NAC, CAT or TROLOX. Colonies and single cells were counted, and colony formation efficiency was calculated. (D, E) 4E KO NT2197 cells expressing empty vector (EV) or 4EBP1^{AA} were injected in the mammary fat pad of NOD *SCID* gamma mice. Tumors were harvested, photographed (D) and weighed (E). n=10 mice for EV NT2197 cells and 8 mice for 4EBP1^{AA} NT2197 cells. (F, G) WT NT2197, shGFP and shAcaca 4E KO NT2197 tumors were lysed and dityrosine levels were determined by immunoblot (F) and quantified (G). n=5 tissues for shAcaca and WT tumors and n=4 tissues for shGFP tumors. Representative immunoblots of two independent experiments are shown (F). Data are shown as the mean ± SD. Statistics: unpaired one-sided Student's *t* test (A, B, C, E), one way ANOVA (G); n=3 independent experiments for A, B, C. Source data are provided as a Source Data file.

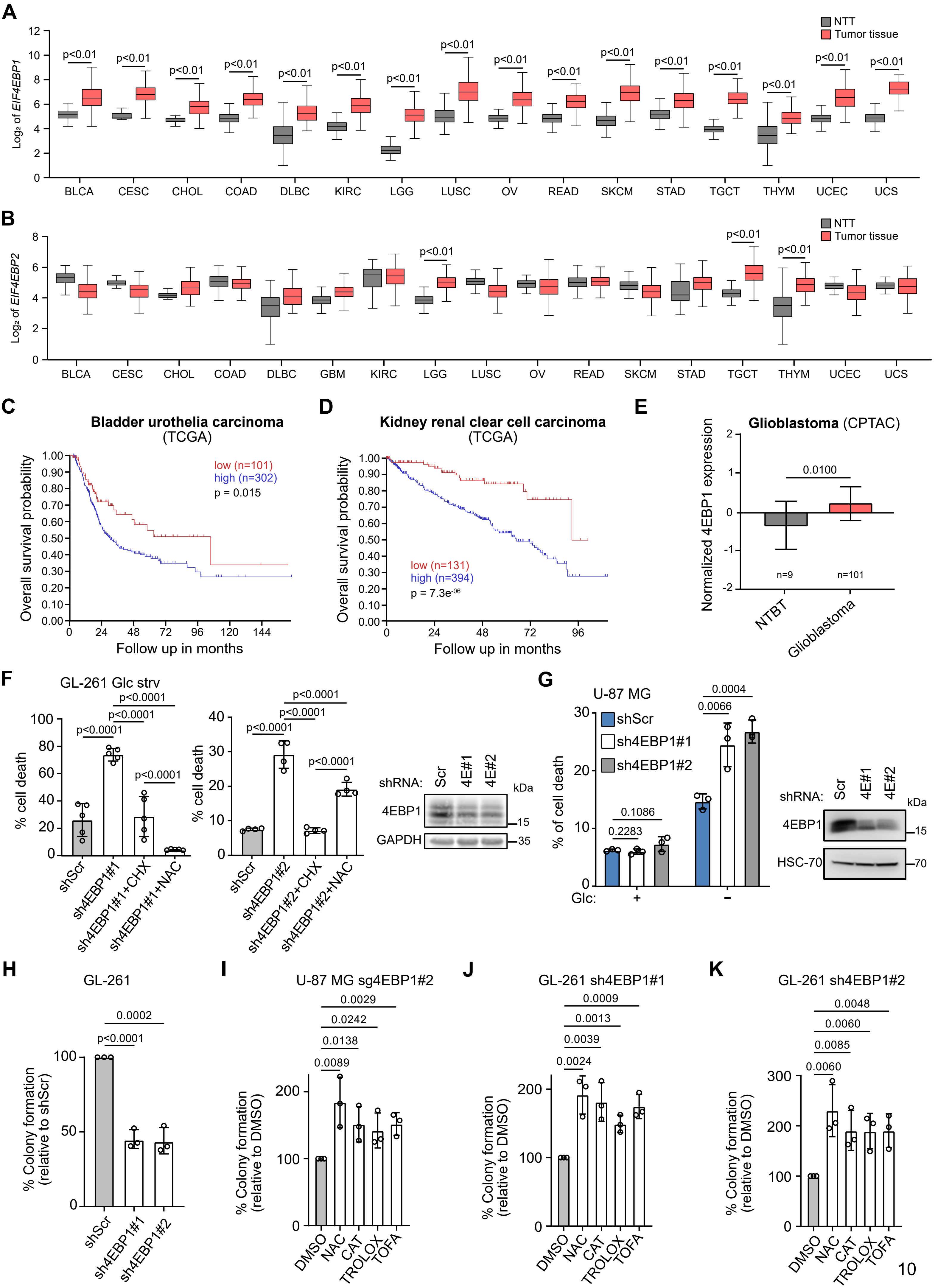
A
B
C
Bladder urothelia carcinoma (TCGA)
D
Kidney renal clear cell carcinoma (TCGA)
E
Glioblastoma (CPTAC)
F
GL-261 Glc strv
G
U-87 MG
H
GL-261
I
U-87 MG sg4EBP1#2
J
GL-261 sh4EBP1#1
K
GL-261 sh4EBP1#2

Supplementary Figure 7. Clinical relevance of *EIF4EBP1* expression in cancer patients and contribution of 4EBP1 to survival of glioma cells under glucose deprivation

(A, B) Expression levels of *EIF4EBP1* (A) and *EIF4EBP2* (B) in the indicated tumor entities and corresponding non-tumorigenic tissues (NTT) from TCGA. p values were calculated using an unpaired and two-tailed parametric *t* test. (C, D) Kaplan-Meier survival estimates of overall survival of bladder urothelial carcinoma (C) and kidney renal clear cell carcinoma (D) patients stratified by their *EIF4EBP1* mRNA levels (cut off first quartile) in the indicated cohorts. p values were calculated using a log rank test. (E) Expression levels of 4EBP1 protein in non-tumorigenic brain tissue (NTBT) and glioblastoma tissues from CPTAC GBM proteomic data. Data are shown as boxplots with medians, interquartile ranges and lower/upper whiskers in. p value was calculated using a two-tailed Mann-Whitney test. (F) The indicated cells were glucose starved (Glc strv) with or without cycloheximide (CHX) or NAC for 48 hrs. Cell death was measured by PI staining and flow cytometry. The levels of the indicated proteins were analyzed by immunoblotting. (G) The indicated cells were grown in complete medium or glucose (Glc) starved for 48 hrs. Cell death and protein levels were analyzed as in (F). (H) The indicated cells were grown in soft agar for 21 days. Colonies and single cells were counted, and colony formation efficiency was calculated and normalized to shScr. (I, J, K) The indicated cells were grown in soft agar for 21 days and treated with DMSO, NAC, CAT, TROLOX or TOFA. Colonies and single cells were counted, and colony formation efficiency was calculated and normalized to DMSO. Data are the mean ± SD for F, G, H, I, J, K. Statistics: one way ANOVA (F), two way ANOVA (G), unpaired one-sided Student's *t* test (H, I, J, K); n=3-4 independent experiments for F, n=3 independent experiments for G, H, I, J, K. Source data are provided as a Source Data file.

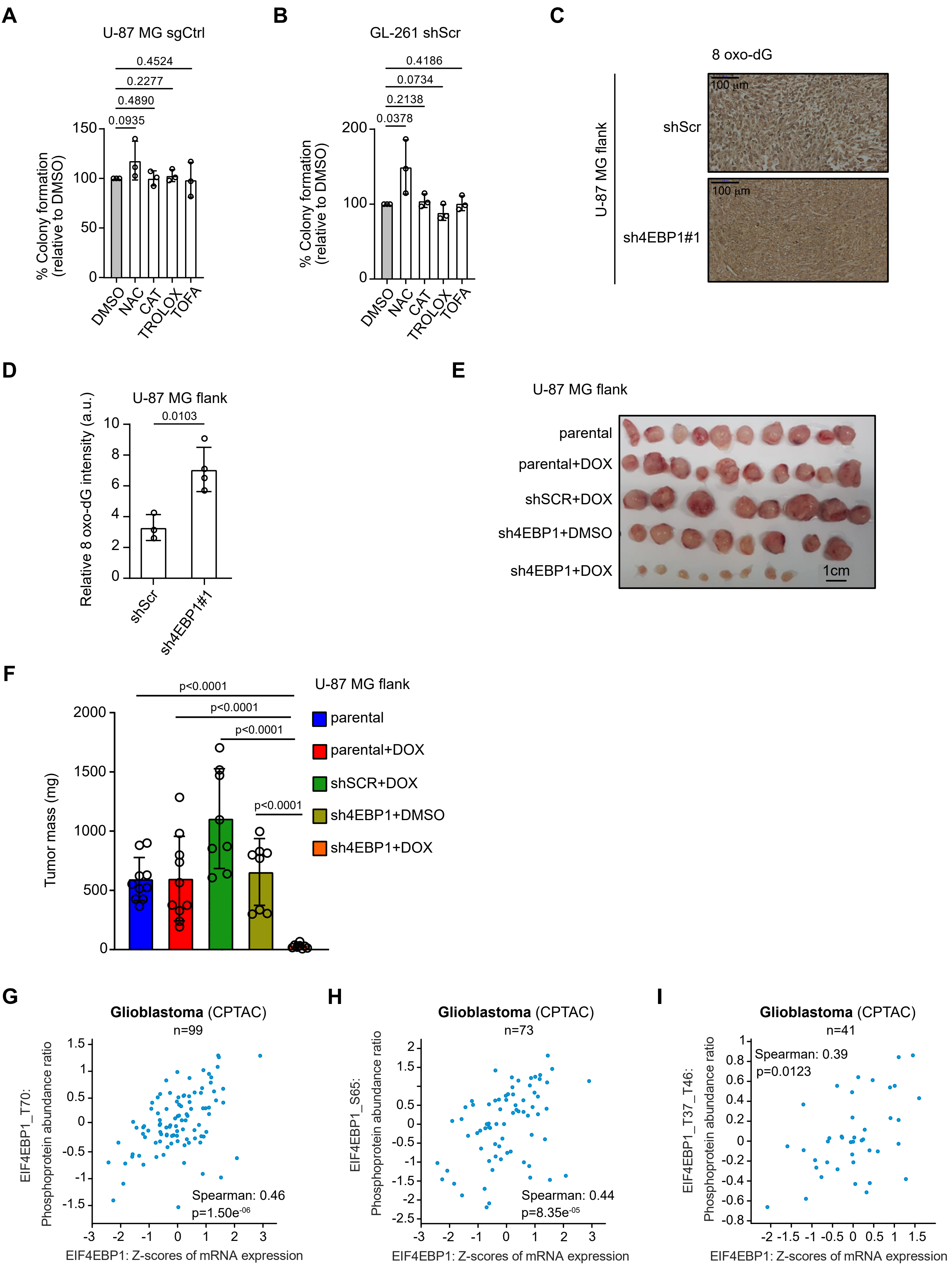
A
U-87 MG sgCtrl
0.4524
0.2277
0.4890
0.0935
% Colony formation (relative to DMSO)
DMSO
NAC
CAT
TROLOX
TOFA
B
GL-261 shScr
0.4186
0.0734
0.2138
0.0378
C
8 oxo-dG
U-87 MG flank
shScr
sh4EBP1#1
100 µm
D
U-87 MG flank
0.0103
Relative 8 oxo-dG intensity (a.u.)
shScr
sh4EBP1#1
E
U-87 MG flank
parental
parental+DOX
shSCR+DOX
sh4EBP1+DMSO
sh4EBP1+DOX
1cm
F
U-87 MG flank
p<0.0001
Tumor mass (mg)
parental
parental+DOX
shSCR+DOX
sh4EBP1+DMSO
sh4EBP1+DOX
G
Glioblastoma (CPTAC)
n=99
EIF4EBP1_T70: Phosphoprotein abundance ratio
Spearman: 0.46
p=1.50e-06
EIF4EBP1: Z-scores of mRNA expression
H
Glioblastoma (CPTAC)
n=73
EIF4EBP1_S65: Phosphoprotein abundance ratio
Spearman: 0.44
p=8.35e-05
EIF4EBP1: Z-scores of mRNA expression
I
Glioblastoma (CPTAC)
n=41
EIF4EBP1_T37_T46: Phosphoprotein abundance ratio
Spearman: 0.39
p=0.0123
EIF4EBP1: Z-scores of mRNA expression

Supplementary Figure 8. 4EBP1 restricts DNA oxidation in glioma tumors and *EIF4EBP1* expression correlates with levels of phospho-4EBP1 in glioblastoma

(A, B) The indicated cells were grown in soft agar for 21 days and treated with DMSO, NAC, CAT, TROLOX or TOFA. Colonies and single cells were counted, and colony formation efficiency was calculated and normalized to DMSO. n=3 independent experiments. (C, D) Levels of 8-Oxo-2'-deoxyguanosine (8-oxo-dG) were determined in shScr and sh4EBP1#1 U-87 MG tumors by IHC (C) and quantified (D). n=3 tissues for shScr tumors and 4 tissues for sh4EBP1 tumors. (E, F) Inducible shRNA U87 cells were injected to the flanks of NOD *SCID* mice. When tumors reached 100 mm^3, doxycycline was added to the drinking water. When the control tumors reached 1,400 mm^3. All tumors were harvested and weighed (J). n=10 mice for the parental cell lines and n=8 mice for the shSCR and sh4EBP1 cell lines. (G-I) Expression levels of *EIF4EBP1* mRNA in glioblastoma patient samples plotted against the expression levels of phospho-4EBP1^{T70} (G), phospho-4EBP1^{S65} (H) or phospho-4EBP1^{T37,T46} (I) using CPTAC GBM data. Co-expression levels were quantified by calculating the Spearman's rank correlation coefficient. Data are shown as the mean ± SD for A, B, D, F. Statistics: unpaired one-sided Student's *t* test (A, B, D), one way ANOVA (F).

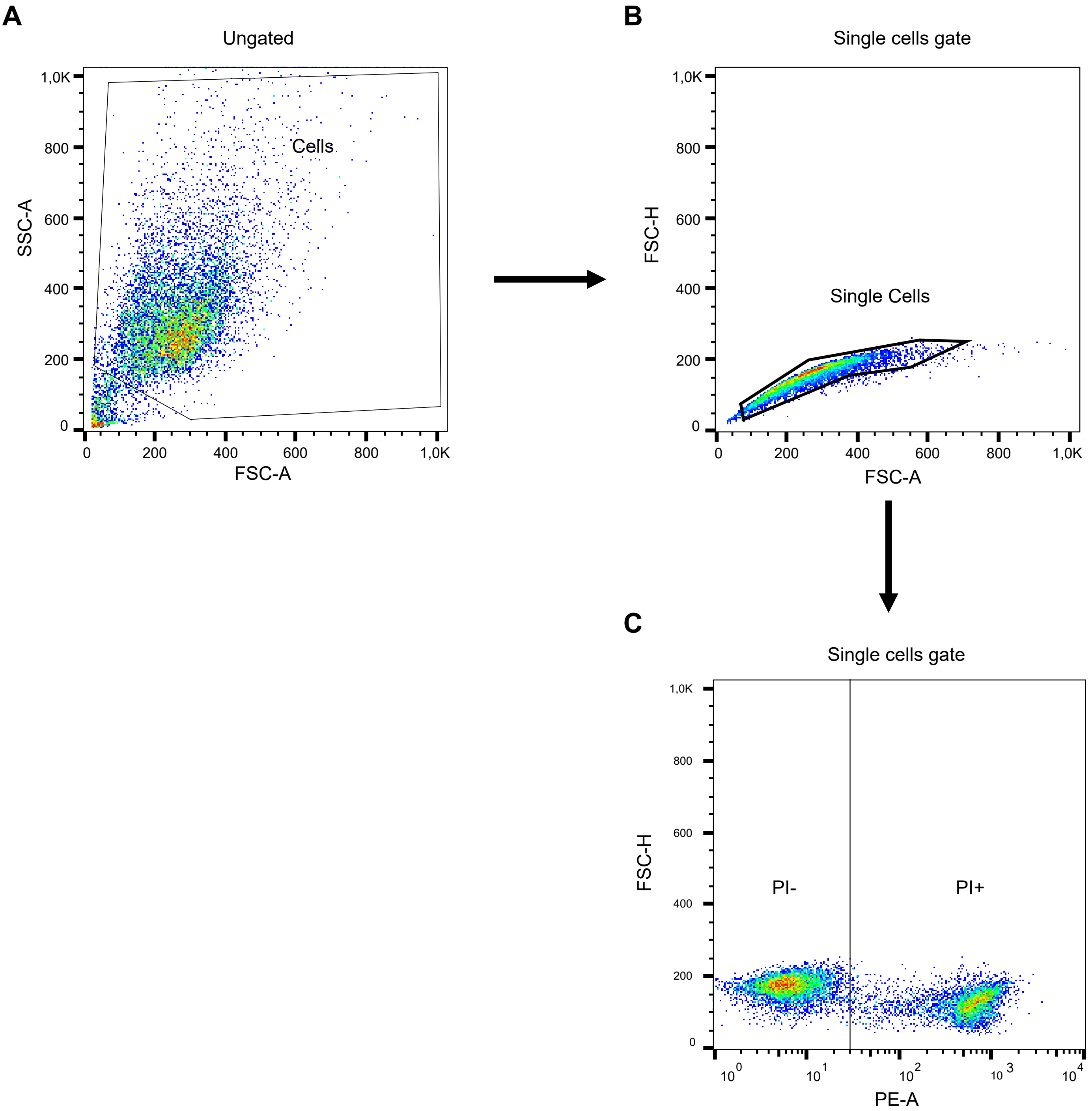

Supplementary Figure 9. Flow cytometry gating strategy

Gating hierarchy of a representative sample stained with PI. (A) Cells were gated in FSC-A versus SSC-A to remove cell debris. (B) Single cells were gated in FSC-A versus FSC-H. (C) Two distinct groups (PI- and PI+ cells) were identified in PE-A versus FSC-H.

Supplementary table 1: List of siRNA sequences

siRNA label and target gene	siRNA sequence
Dharmacon – Horizon Discovery	
siGENOME Non-targeting Pool#1	5’- UGGUUUACAUGUCGACUAA -3’
	5’- UAAGGCUAUGAAGAGAUAC -3’
	5’- AUGUAUUGGCCUGUAUUAG -3’
	5’- AUGAACGUGAAUUGCUCAA -3’
siGENOME mouse Eif4ebp1 Smart Pool	5’- GAACCAGGAUUAUCUAUGA -3’
	5’- CAAAGGACCUGCCAGCCAU -3’
	5’- GCGAUGAGCCUCCCAUGCA -3’
	5’- CCAGCAGCCCGGAAGAUAA -3’
siGENOME mouse Eif4ebp2 Smart Pool	5’- GGGAGGAACACGAAUCAUU -3’
	5’- UGAACAAUCAUGACAGGAA -3’
	5’- GCACCGUGGCUAUCAGCGA -3’
	5’- GUUGGACCGUCGCAAUUCU -3’
siGENOME human and mouse EIF4E siRNA#1	5’- CAUAUCCAGUUGUCUAGUA -3’
siGENOME human and mouse EIF4E siRNA#2	5’- GUGAUAAGAUAGCAAUAUG -3’
siGENOME human ACACA siRNA#1	5’- CAGCAAACCUGGAUUCUGA -3’
siGENOME human ACACA siRNA#2	5’- GCAAUUAGAUUCGUUGUCA -3’
siGENOME mouse Acaca siRNA#1	5’- AGAUAGAAUCAUCGAGUUU -3’
siGENOME mouse Acaca siRNA#2	5’- GGAUCAAGGAUAUUCGUAU -3’
ON-TARGETplus human FASN siRNA#1	5’- GAAGCACAUUGGCAAAGUC -3’
ON-TARGETplus human FASN siRNA#2	5’- CUUCCGAGAUUCCAUCCUA -3’
Sigma Aldrich	
mouse Fasn siRNA#1, sense	5’- GUCAGAUCCUGGAACGAGA[dT][dT] -3’
mouse Fasn siRNA#1, antisense	5’- UCUCGUUCCAGGAUCUGAC[dT][dT] -3’
mouse Fasn siRNA#2, sense	5’- GUAAUGCUGGCCAAACUAA[dT][dT] -3’
mouse Fasn siRNA#2, antisense	5’- UUAGUUUGGCCAGCAUUAC[dT][dT] -3’

Supplementary table 2: List of CRISPRi sgRNA sequences

sgRNA label and target gene	sgRNA sequence
sgRNA control, non-targeting	5’- GACCGCGCCAAACGTGCCCTGACGG-3’
sgRNA human *EIF4EBP1* #1	5’- GGACATGGTCTCCTGTGCGCG -3’
sgRNA human *EIF4EBP1* #2	5’- GTGCGCTGCACCCGCGAACCG -3’
sgRNA mouse *ACACA*	5’- GCCCAGCACATCTCGGCGCAG -3’

Supplementary table 3: List of RT-qPCR primers

Primers target gene	sequence
ACACA	FW: 5’- GCTGGTCCACATGAACAGG -3’<br>RV: 5’- GCCTTCTGGATATTCAGGACTTT -3’
EAP1	FW: 5’- CAGCCGCTACTCACAAATC -3’<br>RV: 5’- GCTTTCTTTATTGTTACCGCTC -3’
ACT1	FW: 5’- CCAGAAGCTTTGTTCCATCC -3’<br>RV: 5’- CGGACATAACGATGTTACCG -3’
EIF4EBP1	FW: 5’- AGCCCTTCCAGTGATGAGC -3’<br>RV: 5’- TGTCCATCTCAAACTGTGACTCTT -3’
Eif4ebp1	FW: 5’- CTAGCCCTACCAGCGATGAG -3’<br>RV: 5’- CCTGGTATGAGGCCTGAATG -3’
GusB	FW: 5’- GTTTTTGATCCAGACCCAGATG -3’<br>RV: 5’- GCCCATTATTCAGAGCGAGTA -3’
PPIA	FW: 5’- TTATTTGGGTTGCTCCCTTC -3’<br>RV: 5’- AAGTGTGCCAAATCTGCAAG -3’
β-actin	FW: 5’- TCCCCCAACTTGAGATGTATG -3’<br>RV: 5’- ACTGGTCTCAAGTCAGTGTACAGG -3’
L32	FW: 5’- GCACACTGACTACAGCCTTGA -3’<br>RV: 5’- TACCCAGGTTTGGAGGTGTG -3’

Supplementary table 4: List of shRNA sequences

shRNA label and target gene	shRNA sequence
scramble shRNA, for Tet-pLKO-puro	5'- CCGGTCCTAAGGTTAAGTCGCCCTCGCTCG AGCGAGGGCGACTTAACCTTAGGTTTTTG -3'
human *EIF4EBP1* shRNA#1, for Tet-pLKO-puro	5'- CCGGGCCAGGCCTTATGAAAGTGATCTCGA GATCACTTTCATAAGGCCTGGCTTTTTG -3'
human *EIF4EBP1* shRNA#2, for Tet-pLKO-puro	5'- CCGGCGGTGAAGAGTCACAGTTTGACTCGA GTCAAACTGTGACTCTTCACCGTTTTTG -3'
mouse *ACACA* shRNA, for pLKO.1-neo	5'- CCGGAGATAGAATCATCGAGTTTCTCGAGA AACTCGATGATTCTATCTTTTTTG -3'

Mice used in experiments

Figure	Species	Strain	Sex	Number	Age	Ethical approval
5E&F	mice	NOD *SCID* gamma	F	22	8W	IL59082019E
5G&H	mice	NOD *SCID* gamma	M	22	7W	IL59082019E
5I&J	mice	NOD *SCID* gamma	F	18	8W	IL59082019E
S6D&E	mice	NOD *SCID* gamma	F	18	7W	IL59082019E
6F	mice	NOD *SCID* (Jackson Labs)	M	35	8W	IL80122015E
6G, S8I&J	mice	NOD *SCID* gamma	F	44	7W	IL59082019E
6H	mice	NOD *SCID* gamma	M	15	6W	IL34062016E
6I	mice	C57BL/6J (Envigo)	F	17	8W	IL59082019E
6J	mice	C57BL/6J (Envigo)	F	17	8W	IL59082019E

NOD *SCID* gamma mice (NOD.Cg-Prkdcscid Il2rgtm1Wjl/SzJ), Jackson Labs, Bar Harbor ME, USA.

Key resources table

REAGENT or RESOURCE	SOURCE	IDENTIFIER
Antibodies / dilution		
4E-BP1 / 1:1000	Cell Signaling Technology	Cat#9644; RRID: AB_2097841
4E-BP2 / 1:1000	Cell Signaling Technology	Cat#2845; RRID: AB_10699019
ACC1 (Acetyl-CoA Carboxylase 1) / 1:500	Cell Signaling Technology	Cat#4190; RRID: AB_796746
ACC2 / 1:1000	Cell Signaling Technology	Cat#8578; RRID: AB_10949898
Anti-mouse IgG / 1:10000	Cell Signaling Technology	Cat#7076; RRID: AB_330924
Anti-rabbit IgG / 1:10000	Cell Signaling Technology	Cat#7074; RRID: AB_2099233
Anti-β-Actin / 1:1000	Sigma Aldrich	Cat#A2228; RRID: AB_476697
GAPDH / 1:2000	Cell Signaling Technology	Cat#2118; RRID: AB_561053
LC3B / 1:1000	Cell Signaling Technology	Cat#2775; RRID: AB_915950
Phospho-Acetyl-CoA Carboxylase (S79) / 1:500	Cell Signaling Technology	Cat#3661; RRID: AB_330337
Phospho-AMPKalpha (T172) / 1:500	Cell Signaling Technology	Cat#2535; RRID: AB_331250
Phospho-S6 Ribosomal Protein (S240/244) / 1:1000	Cell Signaling Technology	CAT#2215; RRID: AB_331682
Vinculin / 1:1000	Cell Signaling Technology	Cat#4650; RRID: AB_10559207

Phospho-ULK1 (S555) / 1:1000	Cell Signaling Technology	Cat#5869; RRID: AB_10707365
eIF4E / 1:1000	Cell Signaling Technology	Cat#9742; RRID: AB_823488
FASN (Fatty acid synthase) / 1:1000	Cell Signaling Technology	Cat#3180; RRID: AB_2100796
ACLY / 1:1000	Cell Signaling Technology	Cat#13390; RRID: AB_2798203
AMPKalpha / 1:1000	Cell Signaling Technology	Cat#2532; RRID: AB_330331
Dityrosine / 1:1000	AdipoGene	Cat#JAI-MDT-020P
8-hydroxy-2'-deoxyguanosine / 1:250	R&D Systems	Cat#4345-MC-050
Mouse specific HRP/DAB / 1:200	Abcam	Cat#ab64259
IRDye® 800CW Goat anti-Mouse IgG Secondary Antibody / 1:10000	LI-COR Bioscience	Cat#925-32210; RRID: AB_2687825
IRDye® 800CW Goat anti-Rabbit IgG Secondary Antibody / 1:10000	LI-COR Bioscience	Cat#925-32211; RRID: AB_2651127
ULK1 / 1:1000	Cell Signaling Technology	Cat#8054; RRID: AB_11178668
HSC-70 / 1:5000	Santa Cruz	Cat#sc-7298; RRID: AB_627761
Mouse anti-HA-tag (F-7) / 1:1000	Santa Cruz	Cat#sc-7392; RRID: AB_2894930
Streptavidin-HRP / 1:10000	ABCAM	Cat#ab59653
Bacterial and Virus Strains		
Competent *E.coli* Stbl3	This study	N/A
Chemicals, Peptides, and Recombinant Proteins		
N-acetyl-cysteine (NAC)	Sigma-Aldrich	Cat#A7250
Catalase-polyethylene glycol (CAT)	Sigma-Aldrich	Cat#C4963
6-hydroxy-2,5,7,8-tetramethylchroman-2-carboxylic acid (Trolox)	Sigma-Aldrich	Cat#238813
5-(Tetradecyloxy)-2-furoic acid (TOFA)	Sigma-Aldrich	Cat#T6575
Dimethyl sulfoxide (DMSO)	Sigma-Aldrich	Cat#D8418
Cycloheximide (CHX)	Sigma-Aldrich	Cat#01810
Gemcitabine hydrochloride	Sigma-Aldrich	Cat#G6423
Crystal violet	Sigma-Aldrich	Cat#C0775
Rapamycin	Selleckchem	Cat#S1039
Ku-0063794	Selleckchem	Cat#S1226
4EGI-1	Selleckchem	Cat#S7369
Proteinase K	Sigma-Aldrich	Cat#3115887001
Doxycycline hydrochloride (DOX)	Santa Cruz	Cat#sc-337691
Critical Commercial Assays		
Pierce™ BCA Protein Assay Kit	ThermoFisher Scientific	Cat#23225
NADP/NADPH-Glo™	Promega	Cat#G9081
NAD/NADH-Glo™	Promega	Cat#G9071
GSH/GSSG-Glo™	Promega	Cat#V6611

Click-iT™ Protein Reaction Buffer Kit	ThermoFisher Scientific	Cat#C10276
Click-iT™ EdU Cell Proliferation Kit for Imaging, Alexa Fluor™ 488	ThermoFisher Scientific	Cat#C10337
Dual-Luciferase Reporter Assay System	Promega	Cat#E1980
TMRE Mitochondrial Membrane Potential Assay Kit	Cayman Chemical	Cat#701310
Deposited Data		
CPTAC GBM proteomic data	[1]	https://cptac-data-portal.georgetown.edu/cptac/s/S048
TCGA GBM proteomic data	[2]	
**From R^{2}AMC:**		http://r2.amc.nl
FRENCH cohort	[3]	GEO ID: gse106011
TCGA bladder carcinoma		ID: BLCA
TCGA Kidney renal clear cell carcinoma		ID: KIRC
**From the GEPIA website:**		http://gepia.cancer-pku.cn
GTEx across datasets	[4]	
TCGA across datasets	[4]	
**From the Chinese Glioma Genome Atlas website:**		www.cgga.org.cn
CGGA cohort	[5]	
Experimental Models: Cell Lines		
Human: HEK293 (human embryonic kidney)	American Type Culture Collection (ATCC)	Cat#CRL-1573
Human: HEK293-T (SV40 T-antigen containing human embryonic kidney cells)	ATCC	Cat#CRL-3216
Human: U-87 MG (glioblastoma)	ATCC	Cat#HTB-14
Human: HeLa (cervical adenocarcinoma)	ATCC	Cat#CRM-CCL-2
Human: MCF7 (breast cancer)	ATCC	Cat#HTB-22
Human: IMR-32 (neuroblastoma)	Alexander Schramm (University Hospital Essen)	N/A
Human: Kelly (neuroblastoma)	Alexander Schramm (University Hospital Essen)	N/A
Human: Med8a (medulloblastoma)	Pablo Landgraf (University Hospital Cologne, Cologne)	N/A
Human: HD-MB03 (medulloblastoma)	Till Milde (DKFZ, Heidelberg)	N/A
Human: iPSC	TakaraBio	Cat#Y00270
Human: HEK293 shRNA control (shScr)	[6]	N/A
Human: HEK293 shRNAs 4EBP1, 4EBP2 (sh4EBP1/2)	[6]	N/A
Mouse: MEF WT (p53-/-) and (mouse embryonic fibroblast, *Tp53* null)	[6]	N/A
Mouse: MEF 4EBP1/4EBP2 double knockout (DKO) (p53-/-) (*Eif4ebp1*, *Eif4ep2*, *Tp53* null)	[6]	N/A
Mouse: MEF AMPKα knockout (*AMPKα1*, *AMPKα2* null)	[7]	N/A
Mouse: NMuMG-NT2197 control	[8]	N/A

Mouse: NMuMG-NT2197 4EBP1/4EBP2 double knockout (DKO)	[8]	N/A
Mouse: GL-261	Reuven Stein (Tel Aviv University; Israel)	N/A
Mouse: NIH 3T3 K-Ras^{V12}	[9]	N/A
Experimental Models: Organisms/Strains		
S. cerevisiae: BY4742 WT MATα his3Δ1 leu2Δ0 lys2Δ0 ura3Δ0	Andreas Reichert (Heinrich Heine University, Düsseldorf)	N/A
S. cerevisiae: Eap1Δ MATα his3Δ1 leu2Δ0 lys2Δ0 ura3Δ0 eap1Δ::NatMX4	This study	N/A
S. cerevisiae: Caf20Δ MATα his3Δ1 leu2Δ0 lys2Δ0 ura3Δ0 caf20Δ::KanMX4	This study	N/A
S. cerevisiae: Eap1Δ/Caf20Δ MATα his3Δ1 leu2Δ0 lys2Δ0 ura3Δ0 eap1Δ::NatMX4 caf20Δ::KanMX4	This study	N/A
Mouse: NOD *SCID* Gamma *Prkdc^{scid}*	The Jackson Laboratory	RRID:IMSR_JAX:001303
Mouse: C57BL/6J	The Jackson Laboratory	RRID:IMSR_JAX:000664
Oligonucleotides		
siGENOME Non-Targeting (scramble) Pool#1	Dharmacon – Horizon Discovery	Cat#D1206-13-05
siGENOME mouse Eif4ebp1 siRNA Pool	Dharmacon – Horizon Discovery	Cat#D-05861-01
siGENOME mouse Eif4ebp2 siRNA Pool	Dharmacon – Horizon Discovery	Cat#D-044972-01
siGENOME human and mouse EIF4E siRNA#1	Dharmacon – Horizon Discovery	Cat#D-003884-02
siGENOME humanand mouse EIF4E siRNA#2	Dharmacon – Horizon Discovery	Cat#D-003884-03
siGENOME human ACACA siRNA#1	Dharmacon – Horizon Discovery	Cat#D-004551-03
siGENOME human ACACA siRNA#2	Dharmacon – Horizon Discovery	Cat# D-004551-05
siGENOME mouse Acaca siRNA#1	Dharmacon – Horizon Discovery	Cat#D-063938-18
siGENOME mouse Acaca siRNA#2	Dharmacon – Horizon Discovery	Cat#D-063938-20
ON-TARGETplus human FASN siRNA#1	Dharmacon – Horizon Discovery	Cat# J-003954-12-0005
ON-TARGETplus human FASN siRNA#2	Dharmacon – Horizon Discovery	Cat# J-003954-14-0005
mouse Fasn siRNA#1	Sigma Aldrich	Cat#SASI_Mm01_00177854
mouse Fasn siRNA#2	Sigma Aldrich	Cat#SASI_Mm01_00177855
SiRNA sequences, see Table S1		
CRISPRi sgRNA Control	This study	N/A
CRISPRi sgRNA targeting human *EIF4EBP1* #1	This study	N/A
CRISPRi sgRNA targeting human *EIF4EBP1* #2	This study	N/A
CRISPRi sgRNA targeting mouse *Acaca*	This study	N/A
SgRNA sequences, see Table S2		
Primers for qRT-PCR, see Table S3		

Recombinant DNA		
Scramble: pLKO.1-puro scramble shRNA	Addgene	Cat#1864
sh4EBP1#1: *EIF4EBP1* MISSION shRNA (human, pLKO.1-puro)	Sigma Aldrich	TRCN0000040203
sh4EBP1#2: *EIF4EBP1* MISSION shRNA (human, pLKO.1-puro)	Sigma Aldrich	TRCN0000298904
sh4EBP1#1: *Eif4ebp1* MISSION shRNA (mouse, pLKO.1-puro)	Sigma Aldrich	TRCN0000075610
sh4EBP1#2: *Eif4ebp1* MISSION shRNA (mouse, pLKO.1-puro)	Sigma Aldrich	TRCN0000348615
pLKO.1-neo	Addgene	Cat#13425
shGFP: GFP shRNA (pLKO.1-neo)	Addgene	Cat#72571
shAcaca: *Acaca* shRNA (mouse, pLKO.1-neo)	This study	N/A
Tet-pLKO-puro	Addgene	Cat#21915
Scramble inducible: Tet-pLKO-puro Non-Mammalian shRNA Control	This study	N/A
ish4EBP1#1: *EIF4EBP1* shRNA#1 (human, Tet-pLKO-puro)	This study	N/A
ish4EBP1#2: *EIF4EBP1* shRNA#2 (human, Tet-pLKO-puro)	This study	N/A
gRNA-dCas9-KRAB GFP	Addgene	Cat#71237
sgCtrl: negative control sgRNA (gRNA-dCas9-KRAB GFP)	This study	N/A
sgAcaca: *Acaca* sgRNA (mouse, gRNA-dCas9-KRAB GFP)	This study	N/A
sg4EBP1#1: *EIF4EBP1* sgRNA#1 (human, gRNA-dCas9-KRAB GFP)	This study	N/A
sg4EBP1#2: *EIF4EBP1* sgRNA#2 (human, gRNA-dCas9-KRAB GFP)	This study	N/A
pLJM1	Addgene	Cat#91980
pLJM1-4EBP1 (T37A/T46A) [4EBP1^{AA}]	This study	N/A
pLJM1-4EBP1AA (Y54A/L59A) [4EBP1$^{AA, YL}$]	This study	N/A
pMSCV puro	Clontech	Cat#K1062-1
pMSCV puro-4EBP1 (T37A/T46A) [4EBP1^{AA}]	[10]	N/A
psPAX2	Addgene	Cat#12260
pMD2.G	Addgene	Cat#12259
pCDNA3.1	ThermoFisher Scientific	Cat# V79020
pCDNA3.1-ACC1-HA	This study	N/A
pGL3 control	Promega	Cat#E1741
pGL3-ACACA 5'UTR (5'UTR of human *ACACA* isoform 3)	This study	N/A
pUb-ACC1-HA	This study	N/A
pUb-UTR-ACC1-HA	This study	N/A
pRL null *Renilla* Luciferase plasmid	Promega	Cat#E2271
Software and Algorithms		
R2 Genomic Analysis Visualization Platform		http://r2.amc.nl
GEPIA website	[4]	http://gepia.cancer-pku.cn
GraphPad Prism version 7.04 and 8.0.2	GraphPad Software	
cBioportal	[11, 12]	https://www.cbioportal.org/

Other		
Calfectin^{TM} Mammalian Cell Transfection Reagent	SignaGen	Cat#SL100478
siLentFect^{TM} Lipid Reagent for RNAi	BioRad	Cat#1703362
CM-H2DCFDA	ThermoFisher Scientific	Cat#C6827
5'-Ethynyl-2'-deoxyuridine (EdU)	ThermoFisher Scientific	Cat#A10044
Azidohomoalanine (AHA)	ThermoFisher Scientific	Cat#C10102
[1-^{14}C]-acetate	Perkin Elmer	Cat#NEC084A001MC
Palmitic Acid, [9,10-^{3}H(N)]	Perkin Elmer	NET043001MC
MitoTracker™ Green FM	ThermoFisher Scientific	Cat#M7514

References

1. Wang, L.B. *et al.* Proteogenomic and metabolomic characterization of human glioblastoma. *Cancer Cell* **39**, 509-528 e520 (2021).
2. Brennan, C.W. *et al.* The somatic genomic landscape of glioblastoma. *Cell* **155**, 462-477 (2013).
3. Gravendeel, L.A. *et al.* Intrinsic gene expression profiles of gliomas are a better predictor of survival than histology. *Cancer Res* **69**, 9065-9072 (2009).
4. Tang, Z. *et al.* GEPIA: a web server for cancer and normal gene expression profiling and interactive analyses. *Nucleic Acids Res* **45**, W98-W102 (2017).
5. Zhao, Z. *et al.* Chinese Glioma Genome Atlas (CGGA): A Comprehensive Resource with Functional Genomic Data from Chinese Glioma Patients. *Genomics Proteomics Bioinformatics* **19**, 1-12 (2021).
6. Dowling, R.J.O. *et al.* mTORC1-mediated cell proliferation, but not cell growth, controlled by the 4E-BPs. *Science (New York, N.Y.)* **328**, 1172-1176 (2010).
7. Laderoute, K.R. *et al.* 5'-AMP-activated protein kinase (AMPK) is induced by low-oxygen and glucose deprivation conditions found in solid-tumor microenvironments. *Molecular and cellular biology* **26**, 5336-5347 (2006).
8. Hulea, L. *et al.* Translational and HIF-1alpha-Dependent Metabolic Reprogramming Underpin Metabolic Plasticity and Responses to Kinase Inhibitors and Biguanides. *Cell Metab* **28**, 817-832 e818 (2018).
9. Leprivier, G. *et al.* The eEF2 kinase confers resistance to nutrient deprivation by blocking translation elongation. *Cell* **153**, 1064-1079 (2013).

10. Goldsmith, A.M. *et al.* Transforming growth factor-beta induces airway smooth muscle hypertrophy. *Am J Respir Cell Mol Biol* **34**, 247-254 (2006).
11. Cerami, E. *et al.* The cBio cancer genomics portal: an open platform for exploring multidimensional cancer genomics data. *Cancer Discov* **2**, 401-404 (2012).
12. Gao, J. *et al.* Integrative analysis of complex cancer genomics and clinical profiles using the cBioPortal. *Sci Signal* **6**, pl1 (2013).
