# Supplementary material for: mTORC1 regulates cell survival under glucose starvation through 4EBP1/2-mediated translational reprogramming of fatty acid metabolism
Source: Nat Commun. 2024 May 14;15:4083. doi: 10.1038/s41467-024-48386-y (PMC11094136; doi:10.1038/s41467-024-48386-y)
Supplement: Supplementary file 3 — Reporting Summary [file 41467_2024_48386_MOESM3_ESM.pdf]

nature portfolio

Corresponding author(s): Gabriel Leprivier and Barak Rotblat

Last updated by author(s): Mar 28, 2024

Reporting Summary

Nature Portfolio wishes to improve the reproducibility of the work that we publish. This form provides structure for consistency and transparency in reporting. For further information on Nature Portfolio policies, see our Editorial Policies and the Editorial Policy Checklist.

Statistics

For all statistical analyses, confirm that the following items are present in the figure legend, table legend, main text, or Methods section.

n/a	Confirmed	
☐	☒	The exact sample size (*n*) for each experimental group/condition, given as a discrete number and unit of measurement
☐	☒	A statement on whether measurements were taken from distinct samples or whether the same sample was measured repeatedly
☐	☒	The statistical test(s) used AND whether they are one- or two-sided<br>*Only common tests should be described solely by name; describe more complex techniques in the Methods section.*
☒	☐	A description of all covariates tested
☐	☒	A description of any assumptions or corrections, such as tests of normality and adjustment for multiple comparisons
☐	☒	A full description of the statistical parameters including central tendency (e.g. means) or other basic estimates (e.g. regression coefficient) AND variation (e.g. standard deviation) or associated estimates of uncertainty (e.g. confidence intervals)
☐	☒	For null hypothesis testing, the test statistic (e.g. *F*, *t*, *r*) with confidence intervals, effect sizes, degrees of freedom and *P* value noted<br>*Give P values as exact values whenever suitable.*
☒	☐	For Bayesian analysis, information on the choice of priors and Markov chain Monte Carlo settings
☒	☐	For hierarchical and complex designs, identification of the appropriate level for tests and full reporting of outcomes
☒	☐	Estimates of effect sizes (e.g. Cohen's *d*, Pearson's *r*), indicating how they were calculated

Our web collection on statistics for biologists contains articles on many of the points above.

Software and code

Policy information about availability of computer code

Data collection	RT-qPCR data was collected using Bio-Rad CFX384 real-time system. FACS data was collected using CYFLOW CUBE 6. Radioactive data was collected using Tri-Crab 2100tr liquid scintillation analyzer.
Data analysis	GEPIA website, cBioportal, Fiji software version 1.54f, FlowJo 10 software, FCS Express software and GraphPad Prism version 7.04 and 8.0.2 were used.

For manuscripts utilizing custom algorithms or software that are central to the research but not yet described in published literature, software must be made available to editors and reviewers. We strongly encourage code deposition in a community repository (e.g. GitHub). See the Nature Portfolio guidelines for submitting code & software for further information.

Data

Policy information about availability of data

All manuscripts must include a data availability statement. This statement should provide the following information, where applicable:

- Accession codes, unique identifiers, or web links for publicly available datasets
- A description of any restrictions on data availability
- For clinical datasets or third party data, please ensure that the statement adheres to our policy

The mass spectrometry proteomics data have been deposited to the ProteomeXchange Consortium via the PRIDE partner repository with the dataset identifier

PXD040931 [http://www.ebi.ac.uk/pride/archive/projects/PXD040931]. All other datasets generated and analyzed in this study are provided within the manuscript and the accompanying supplementary figures or from the corresponding authors upon reasonable request. Source data are provided with this paper.

Research involving human participants, their data, or biological material

Policy information about studies with human participants or human data. See also policy information about sex, gender (identity/presentation), and sexual orientation and race, ethnicity and racism.

Reporting on sex and gender	N/A
Reporting on race, ethnicity, or other socially relevant groupings	N/A
Population characteristics	N/A
Recruitment	N/A
Ethics oversight	N/A

Note that full information on the approval of the study protocol must also be provided in the manuscript.

Field-specific reporting

Please select the one below that is the best fit for your research. If you are not sure, read the appropriate sections before making your selection.

☒ Life sciences ☐ Behavioural & social sciences ☐ Ecological, evolutionary & environmental sciences

For a reference copy of the document with all sections, see nature.com/documents/nr-reporting-summary-flat.pdf

Life sciences study design

All studies must disclose on these points even when the disclosure is negative.

Sample size	No statistical methods were used to predetermine sample size. Sample sizes were chosen according to standard practices in the relevant field. Sample size for in vivo experiments were chosen from previous publications (PMID: 31081944; 30704052).
Data exclusions	Mice were excluded from xenograft experiments in cases where the mouse was sick, or tumors grew in the wrong tissue, e.g., muscle instead of subcutaneous tissue.
Replication	All experiments were repeated at least twice with similar results.
Randomization	Mice were allocated randomly into groups.
Blinding	At least three blinded independent researchers performed immunohistochemistry analysis. Researchers were blinded when measuring tumor size and weight.

Reporting for specific materials, systems and methods

We require information from authors about some types of materials, experimental systems and methods used in many studies. Here, indicate whether each material, system or method listed is relevant to your study. If you are not sure if a list item applies to your research, read the appropriate section before selecting a response.

Materials & experimental systems

n/a	Involved in the study	
☐	☒	Antibodies
☐	☒	Eukaryotic cell lines
☒	☐	Palaeontology and archaeology
☐	☒	Animals and other organisms
☒	☐	Clinical data
☒	☐	Dual use research of concern
☒	☐	Plants

Methods

n/a	Involved in the study	
☒	☐	ChIP-seq
☐	☒	Flow cytometry
☒	☐	MRI-based neuroimaging

Antibodies

Antibodies used

Anti-4E-BP1 Cell Signaling Technology Cat#9644; RRID: AB_2097841
Anti-4E-BP2 Cell Signaling Technology Cat#2845; RRID: AB_10699019
Anti-ACC1 (Acetyl-CoA Carboxylase 1) Cell Signaling Technology Cat#4190; RRID: AB_796746
Anti-ACC2 Cell Signaling Technology Cat#8578; RRID: AB_10949898
Anti-β-Actin Sigma Aldrich Cat#A2228; RRID: AB_476697
Anti-GAPDH Cell Signaling Technology Cat#2118; RRID: AB_561053
Anti-LC3B Cell Signaling Technology Cat#2775; RRID: AB_915950
Anti-Phospho-Acetyl-CoA Carboxylase (S79) Cell Signaling Technology Cat#3661; RRID: AB_330337
Anti-Phospho-AMPKalpha (T172) Cell Signaling Technology Cat#2535; RRID: AB_331250
Anti-Phospho-S6 Ribosomal Protein (S240/244) Cell Signaling Technology CAT#2215; RRID: AB_331682
Anti-Vinculin Cell Signaling Technology Cat#4650; RRID: AB_10559207
Anti-Phospho-ULK1 (S555) Cell Signaling Technology Cat#5869; RRID: AB_10707365
Anti-eIF4E Cell Signaling Technology Cat#9742; RRID: AB_823488
Anti-FASN (Fatty acid synthase) Cell Signaling Technology Cat#3180; RRID: AB_2100796
Anti-ACLY Cell Signaling Technology Cat#13390; RRID: AB_2798203
Anti-AMPKalpha Cell Signaling Technology Cat#2532; RRID: AB_330331
Anti-ULK1 Cell Signaling Technology Cat#8054; RRID: AB_11178668
Anti-HSC-70 Santa Cruz Cat#sc-7298; RRID: AB_627761
Mouse anti-HA-tag (F-7) Santa Cruz Cat#sc-7392; RRID: AB_2894930
Anti-dityrosine AdipoGen, JAI-MDT-020P; RRID:AB_1106824
Anti-8-hydroxy-2'-deoxyguanosine R&D Systems, 4354-MC-050; RRID:AB_1857195
Anti-mouse IgG, HRP-linked Cell Signaling Technology Cat#7076; RRID: AB_330924
Anti-rabbit IgG, HRP-linked Cell Signaling Technology Cat#7074; RRID: AB_2099233
Biotinylated goat anti-mouse IgG(H+L) Abcam, ab64255; RRID:AB_2757156
IRDye® 800CW Goat anti-Mouse IgG Secondary Antibody LI-COR Bioscience Cat#925-32210; RRID: AB_2687825
IRDye® 800CW Goat anti-Rabbit IgG Secondary Antibody LI-COR Bioscience Cat#925-32211; RRID: AB_2651127

Validation

All commercial antibodies were validated by the manufacturers as indicated on their official websites.
4E-BP1, https://www.cellsignal.com/products/primary-antibodies/4e-bp1-53h11-rabbit-mab/9644;
4E-BP2, https://www.cellsignal.com/products/primary-antibodies/4e-bp2-antibody/2845
ACC1, https://www.cellsignal.com/products/primary-antibodies/acetyl-coa-carboxylase-1-antibody/4190
ACC2, https://www.cellsignal.com/products/primary-antibodies/acetyl-coa-carboxylase-2-d5b9-rabbit-mab/8578
Anti-β-Actin, https://www.sigmaaldrich.com/IL/en/product/sigma/a2228
GAPDH, https://www.cellsignal.com/products/primary-antibodies/gapdh-14c10-rabbit-mab/2118
LC3B, https://www.cellsignal.com/products/primary-antibodies/lc3b-antibody/2775
Phospho-Acetyl-CoA Carboxylase (S79), https://www.cellsignal.com/products/primary-antibodies/phospho-acetyl-coa-carboxylase-ser79-antibody/3661
Phospho-AMPKalpha (T172), https://www.cellsignal.com/products/primary-antibodies/phospho-ampka-thr172-40h9-rabbit-mab/2535
Phospho-S6 Ribosomal Protein (S240/244), https://www.cellsignal.com/products/primary-antibodies/phospho-s6-ribosomal-protein-ser240-244-antibody/2215
Vinculin, https://www.cellsignal.com/products/primary-antibodies/vinculin-antibody/4650
Phospho-ULK1 (S555), https://www.cellsignal.com/products/primary-antibodies/phospho-ulk1-ser555-d1h4-rabbit-mab/5869
eIF4E, https://www.cellsignal.com/products/primary-antibodies/eif4e-antibody/9742
FASN, https://www.cellsignal.com/products/primary-antibodies/fatty-acid-synthase-c20g5-rabbit-mab/3180
ACLY, https://www.cellsignal.com/products/primary-antibodies/atp-citrate-lyase-d1x6p-rabbit-mab/13390
AMPKalpha, https://www.cellsignal.com/products/primary-antibodies/ampka-antibody/2532
Dityrosine, https://adipogen.com/jai-mdt-020p-anti-dityrosine-dt-mab-1c3.html/
8-hydroxy-2'-deoxyguanosine, https://www.rndsystems.com/products/8-oxo-dg-antibody-15a3_4354-mc-050
ULK1, https://www.cellsignal.com/products/primary-antibodies/ulk1-d8h5-rabbit-mab/8054
HSC-70, https://www.scbt.com/p/hsc-70-antibody-b-6?gad_source=1&gclid=CjwKCAiAibeuBhAAEiwAiXBoJKGEgiu8xTksfjMoJ0a4FxzY4Ig0rXDbkKS-QIWKKYOs7mkmIUd8LhoCp1gQAvD_BwE
Mouse anti-HA-tag (F-7), https://www.scbt.com/p/ha-probe-antibody-f-7?requestFrom=search

Eukaryotic cell lines

Policy information about cell lines and Sex and Gender in Research

Cell line source(s)

Human: HEK293 (human embryonic kidney) American Type Culture Collection (ATCC) Cat#CRL-1573
Human: HEK293-T (SV40 T-antigen containing human embryonic kidney cells) ATCC Cat#CRL-3216
Human: U-87 MG (glioblastoma) ATCC Cat#HTB-14
Human: HeLa (cervical adenocarcinoma) ATCC Cat#CRM-CCL-2
Human: MCF7 (breast cancer) ATCC Cat#HTB-22
Human: IMR-32 (neuroblastoma) Alexander Schramm (University Hospital Essen) N/A
Human: Kelly (neuroblastoma) Alexander Schramm (University Hospital Essen) N/A
Human: Med8a (medulloblastoma) Pablo Landgraf (University Hospital Cologne, Cologne) N/A
Human: HD-MB03 (medulloblastoma) Till Milde (DKFZ, Heidelberg) N/A
Human: iPSC TakaraBio Cat#Y00270
Human: HEK293 shRNA control (shScr) Dowling et al, Science 328, 1172-1176 (2010) N/A
Human: HEK293 shRNAs 4EBP1, 4EBP2 (sh4EBP1/2) Dowling et al, Science 328, 1172-1176 (2010) N/A
Mouse: MEF WT (p53-/-) and (mouse embryonic fibroblast, Tp53 null) Dowling et al, Science 328, 1172-1176 (2010) N/A

	MEF 4EBP1/4EBP2 double knockout (DKO) (p53-/-) (Eif4ebp1, Eif4ep2, Tp53 null) Dowling et al, Science 328, 1172-1176 (2010) N/A<br>Mouse: NMuMG-NT2197 control Hulea et al, Cell Metab 28, 817-832 e818 (2018) N/A<br>Mouse: NMuMG-NT2197 4EBP1/4EBP2 double knockout (DKO) Hulea et al, Cell Metab 28, 817-832 e818 (2018) N/A<br>Mouse: GL-261 Reuven Stein (Tel Aviv University; Israel) N/A<br>Mouse: NIH 3T3 K-RasV12 Leprivier et al, Cell 153, 1064-1079 (2013) N/A
Authentication	HEK293 were authenticated by STR profiling. HEK293-T were authenticated by STR profiling. U-87 MG were authenticated by STR profiling. HeLa were authenticated by STR profiling. MCF7 were authenticated by STR profiling. IMR-32 were authenticated by STR profiling. HEK293 shRNA control (shScr) were authenticated by STR profiling. HEK293 shRNAs 4EBP1, 4EBP2 (sh4EBP1/2) were authenticated by STR profiling. Kelly were authenticated by STR profiling. Med8a were authenticated by STR profiling. HD-MB03 were authenticated by STR profiling.
Mycoplasma contamination	All cell lines were tested negative for mycoplasma.
Commonly misidentified lines<br>(See ICLAC register)	No misidentified cell lines were used.

Animals and other research organisms

Policy information about studies involving animals; ARRIVE guidelines recommended for reporting animal research, and Sex and Gender in Research

Laboratory animals

Figure	Species	Strain	Sex	Age
5E&F	mice	NOD SCID gamma	F	8W
5G&H	mice	NOD SCID gamma	M	7W
5I&J	mice	NOD SCID gamma	F	8W
S6D&E	mice	NOD SCID gamma	F	7W
6F	mice	NOD SCID	M	8W
6G, S8I&J	mice	NOD SCID gamma	F	7W
6H	mice	NOD SCID gamma	M	6W
6I	mice	C57BL/6J	F	8W
6J	mice	C57BL/6J	F	18W

Wild animals	No wild animals were used in the study.
Reporting on sex	Sex was not considered as an experimental factor in this study except in the case of transformed mammary gland models (NMuMG-NT2197 derivative cell lines) where female mice were used.
Field-collected samples	No field-collected samples were used in this study.
Ethics oversight	All mouse work was performed in accordance with the institutional animal care use committee and relevant guidelines at the Ben-Gurion University, with protocols 34-06-2016, 35-06-2016 and 59-08-2019E.

Note that full information on the approval of the study protocol must also be provided in the manuscript.

Plants

Seed stocks	N/A
Novel plant genotypes	N/A
Authentication	N/A

Flow Cytometry

Plots

Confirm that:

☒ The axis labels state the marker and fluorochrome used (e.g. CD4-FITC).

☒ The axis scales are clearly visible. Include numbers along axes only for bottom left plot of group (a 'group' is an analysis of identical markers).

☒ All plots are contour plots with outliers or pseudocolor plots.

☒ A numerical value for number of cells or percentage (with statistics) is provided.

Methodology

Sample preparation	Attached and detached cells were harvested, centrifuged and resuspended in PBS containing 1 µg/ml propidium iodide. The source of the cells is described above in the "Cell line source(s)" section.
Instrument	CytoFLEX flow cytometer (Beckmann Coulter).
Software	FACS data was collected using CYFLOW CUBE 6 and analyzed using FlowJo 10 software.
Cell population abundance	Before sorting, NT2197 4E KO sgCtrl and sgAcaca cells contained 15-30% GFP positive cells, U-87 MG sgCtrl and sg4EBP1 cells contained 50-60% GFP positive cells. After sorting, the GFP positive cell population was close to 100% for all cell lines. Cells were routinely checked for GFP positivity over passages.
Gating strategy	The gating strategy was as follows: 1) The initial cell population was selected through FSC/SSC gates, where debris and dead cells with lower forward scatter were excluded; 2) Single cells were selected on a plot of FSC-A versus FSC-H, excluding doublets and clumps; 3) The density plot was segmented into two areas through PE-A (PI signal) versus FSC-H to identify cells that were either negative or positive for PI.

☒ Tick this box to confirm that a figure exemplifying the gating strategy is provided in the Supplementary Information.
